# Engineered stem cell exosomes for oral and maxillofacial wound healing

**DOI:** 10.3389/fbioe.2022.1038261

**Published:** 2022-10-24

**Authors:** Ming Hao, MengNa Duan, Zhijing Yang, Hengzong Zhou, Shuangji Li, Jingcheng Xiang, Han Wu, Huimin Liu, Lu Chang, Dongxu Wang, Weiwei Liu

**Affiliations:** ^1^ Department of Oral and Maxillofacial Surgery, Hospital of Stomatology, Jilin University, Changchun, China; ^2^ Jilin Provincial Key Laboratory of Tooth Development and Bone Remodeling, Hospital of Stomatology, Jilin University, Changchun, China; ^3^ Department of Prosthodontics, Hospital of Stomatology, Jilin University, Changchun, Jilin, China; ^4^ Laboratory Animal Center, College of Animal Science, Jilin University, Changchun, China

**Keywords:** engineered, exosomes, stem cells, oral and maxillofacial surgery, wound healing

## Abstract

Wound healing of the oral and maxillofacial area affects the quality of life and mental health of the patient; therefore, effective therapies are required to promote wound healing. However, traditional treatment methods have limited efficacy. Exosomes secreted by stem cells used for oral and maxillofacial wound healing have shown outstanding results. Stem cell-derived exosomes possess the regenerative and repair ability of stem cells. Moreover, they are nontumorigenic and have good biosafety. However, the application of natural stem cell exosomes is limited owing to their low yield, impurity, lack of targeting, and low drug delivery rate. Many modification methods have been developed to engineered stem cell exosomes with beneficial properties, such as modifying parent cells and directly processing stem cell exosomes. These methods include coincubation, genetic engineering, electroporation, ultrasound, and artificial synthesis of engineered stem cell exosomes. These engineered stem cell exosomes can cargo nucleic acids, proteins, and small molecules. This gives them anti-inflammatory and cell proliferation regulatory abilities and enables the targeted promotion of efficient soft tissue repair after trauma. Engineered stem cell exosomes can decrease inflammation, promote fibroblast proliferation, and angiogenesis, and decrease scar formation to promote oral and maxillofacial wound healing, including diabetic and burn wounds. Thus, engineered stem cell exosomes are an effective treatment that has the potential for oral and maxillofacial wound healing.

## Introduction

Oral and maxillofacial injuries are common oral and maxillofacial conditions (Durham et al., 2017). Severe oral and maxillofacial skin injury caused by trauma or surgery leads to undesirable healing, such as delayed wound closure and scar formation. This results in physical dysfunction and affects maxillofacial appearance (Tanaka et al., 2021). Wound healing is a complex process involving fibroblast and myofibroblast subpopulations, growth factors, cytokines, and extracellular matrix (ECM) components (Chang et al., 2002; Darby and Hewitson, 2007; Driskell et al., 2013; Dalisson and Barralet, 2019; Hinz and Lagares, 2020; Kim et al., 2021). The physiological wound-healing process can be regulated by external factors and internal biological pathways (Gurtner et al., 2008). Oral and maxillofacial skin injuries, including chronic and infectious wounds and large burns, require positive and effective therapy to promote wound healing (Powers et al., 2016; Hall et al., 2017; Wang Y. et al., 2018; Negut et al., 2018). Proteins, drugs, natural compounds, genes, cells, and bioengineered therapies can be used for wound healing (Cho et al., 2019; Veith et al., 2019). Because of the efficient delivery system, exosomes have potential applications in promoting wound healing.

Exosomes are cup-shaped or spherical bilayer phospholipid membrane structures with a diameter of 40–160 nm released from multivesicular bodies (MVBs) by exocytosis after fusion with the cytoplasmic membrane (Sharma et al., 2010; Sokolova et al., 2011; Kahroba et al., 2019; Kalluri and LeBleu, 2020). The exosome structure enables them to carry various bioactive cargos, such as proteins, nucleic acids (DNA and messenger [mRNA], micro [miRNA], and long non-coding [lncRNA] RNAs), lipids, metabolites, and small molecule drugs (Pascucci et al., 2014; Peng H. et al., 2020; Zhou et al., 2020). The cargos depend on donor cell differentiation and environmental stimulation (Pegtel et al., 2014). Exosomes can be secreted by various cells, such as immune, stem, cardiovascular, nerve, and tumor cells and reticulocytes and platelets (Sokolova et al., 2011). Cell-derived exosomes are widely distributed in the peripheral blood, urine, saliva, sweat, milk, ascites, and amniotic fluid (Admyre et al., 2007; Keller et al., 2007; Dai et al., 2008; Gonzales et al., 2009; Lasser et al., 2011; Wang Q. L. et al., 2018; Wu and Liu, 2018). Their biological functions are exerted by releasing their cargo, such as intercellular signal transduction, cell growth, immune response, and tissue repair and regeneration (Isola and Chen, 2017; Gurunathan et al., 2019).

Exosomes derived from stem cells play a vital role in promoting wound healing by regulating biological processes (Vu et al., 2021). Importantly, blood vessels play an integral role in wound healing by providing oxygen and nutrients for tissues and cells (Eble and Niland, 2009). A previous study has shown that human umbilical cord mesenchymal stem cells (HUC-MSCs) exosomes increase angiogenesis to promote burn wound healing (Zhang et al., 2015b). Moreover, exosomes derived from oral tissue stem cells, urine stem cells, HUC-MSCs, bone marrow mesenchymal stem cells (BMSCs), and mesenchymal stromal cells (MSCs) promote diabetic wound healing and skin regeneration by promoting fibroblast proliferation and migration, angiogenesis, and antioxidant stress (Chen et al., 2018; Shi et al., 2020; Yang et al., 2020; An et al., 2021; Pomatto et al., 2021). Scar formation is commonly seen in wound healing, wherein pathological scar formation affects the physiological functions of hair follicles and sweat glands (Takeo et al., 2015; Monavarian et al., 2019). Stem-cell-derived exosomes conduce to decrease scarring. One study showed that HUC-MSC-derived exosomes inhibit myofibroblast formation, which prevents scar formation (Fang et al., 2016). These findings suggested that stem-cell-derived exosomes positively affected wound healing by promoting cell proliferation and angiogenesis and reducing scarring.

While stem-cell-derived exosomes positively affect wound healing, naturally produced exosomes have limitations that can affect their therapeutic effect, which includes low yield, impurity, and lack of targeting (Shao et al., 2018; Thery et al., 2018) (Table 1). However, the engineering of stem-cell-derived exosomes can improve their yield, purity, targeting, drug delivery, and therapeutic efficacy (Kucuk et al., 2021; Liang et al., 2021). In the present review, we focused on engineering stem cell exosomes, their application in wound healing, and their therapeutic mechanisms.

**TABLE 1:** Comparison of different exosome-extraction methods.

Exosome-extraction method	Advantages	Disadvantages	References
Ultracentrifugation	The gold standard for exosome isolation	Impurity	Thery et al. (2006); Van Deun et al. (2014)
	Feasible for large quantities prepared	Low yield	
	Avoids cross-contamination	High requirements for equipment and technical knowledge	
		Change exosome’s structure and biological function	
		Not conducive to downstream analysis	
Sucrose or iodoxanol gradient centrifugation	Yields high purity	Low yield	Kowal et al. (2016); Chen et al. (2019)
		Need large sample volume	
Coprecipitation	High yield and convenience	Low purity	Rider et al. (2016); Weng et al. (2016)
Dimension exclusion chromatography	Extracellular vesicles can be isolated directly from viscous and complex biological fluids	Low yield	Boing et al. (2014); Muller et al. (2014)
	Rapid; Does not affect the exosome structure and biological function	Impurity	

## Preparation of engineered stem cell exosomes

**FIGURE 1:**
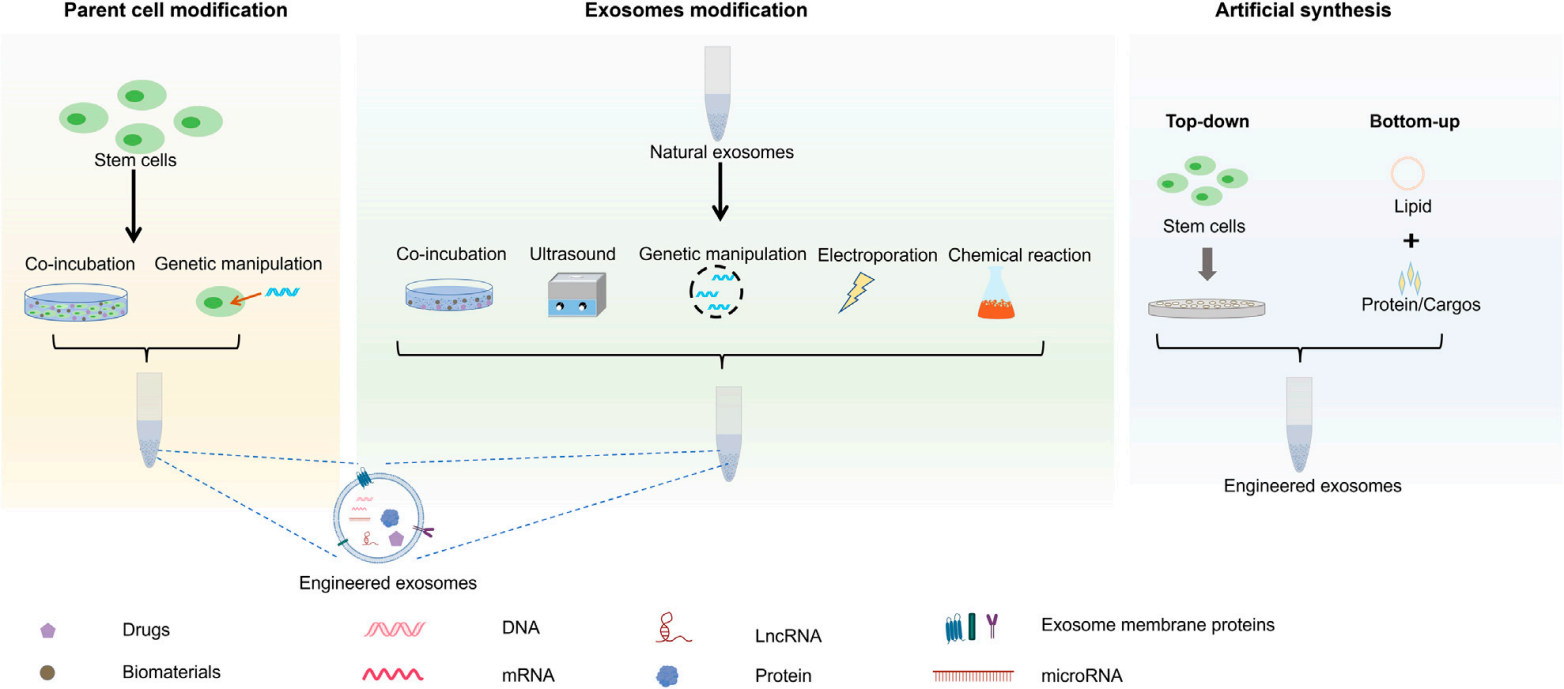
Illustration of the synthesis of engineered stem cell exosomes.

Stem cell exosomes can be engineered with specific functions by indirect, direct, and synthetic preparation methods (Figure 1). These methods, including genetic engineering, co-incubation, parent cell surface modification, and artificial synthesis, have different advantages and limitations (Table 2).

**TABLE 2:** Synthesis classification and characteristics of engineered exosomes.

Classification	Producing method	Cargos	Advantages	Disadvantages	Application	References
Parent cell modification	Transfection	MicroRNA	Wide application	Transfection efficiency variable; Low loading efficiency	Engineered exosomes promote diabetic wound healing by enhancing angiogenesis, fibrogenesis, and re-epithelialization	Huang et al. (2021b)
		LncRNA			HOTAIR overexpressed stem cell exosomes promote the angiogenesis and wound healing of chronic diabetic wounds	Born et al. (2022)
	Co-incubation	Protein	Convenient	Potentially cytotoxic; inefficient loading; Suitable for hydrophilic substances	After IFN-γ stimulation, the overexpression of PD-L1 on exosomes inhibits overactive immune cells and promotes wound healing	Su et al. (2019)
		MicroRNA			Overexpression of miR-1260a in exosomes enhanced osteogenesis and angiogenesis in bone mesenchymal stem cells treated with magnetic nanoparticles combined with static magnetic field	Wu et al. (2021)
Exosome loading	Co-incubation	Curcumin	Enhance the solubility of drugs	Low drug loading rate; Limited application; Suitable for hydrophobic molecules	Curcumin-exosomes possess anti-inflammatory activity and have good therapeutic effect on mice with septic shock induced by lipopolysaccharide (LPS)	Sun et al. (2010)
	Electroporation	MicroRNA	High loading rate; Able to incorporate large compounds	Size-dependent; Exosome aggregation; siRNA loading efficiency variable	Adipose stem cell exosomes overexpressing miR-21-5p promote diabetic wound healing	Kooijmans et al. (2013)
	Ultrasonic	Silver nanoparticles	Stable; high loading rate	Not suitable for thermosensitive compounds	Exosomes carrying AgNP, with antibacterial properties, can accelerate collagen deposition, angiogenesis, and nerve repair to enhance wound healing	Qian et al. (2020)
Exosome surface modification	Genetic engineering	Targeting peptide	Targeting	Need to be characterized	Targeted peptides could be introduced onto the exosome surface by genetically engineered, providing tissue specificity and improving efficacy	Curley et al. (2020)
	Click chemistry	Targeting peptide	Fast reaction time; high specificity	Need to be characterized; A two-step procedure with subsequent purification steps to remove unbound molecules and activate agents	Targeting peptides - Cardiac homing peptides are coupled to cardiac stem cell exosomes to improve their targeting and enhance the uptake of exosomes by cardiac myocytes	Vandergriff et al. (2018)
Artificial synthesis	Top-down	Like parent cells	High purity; large yield	Cargo loading lacks specificity	After passing embryonic stem cells (ES) through micropores, exosome-like microvesicles are obtained, which promote the proliferation of fibroblasts and contribute to tissue recovery or wound-healing processes	Jeong et al. (2014)
	Bottom-up	Protein	Controllable production process; high purity; large yield	Complex operating procedures	By combining bioactive protein with liposome, artificial exosomes with specific co-function were obtained, which reduced synovial hyperplasia and inflammation of rabbit knee joint and had therapeutic effect on rheumatoid arthritis	Martinez-Lostao et al. (2010)

### Parent cell modification

Exosomes carrying nucleic acids and drugs can be derived by treating exosome-secreting cells (Fitts et al., 2019). In the traditional method, the cells are transfected with recombinant viruses or plasmids to obtain exosomes carrying specific genes. Studies show that parent cells transfected with lentiviral vectors produced exosomes carrying miRNA 31-5p (miR-31-5p), which was used to heal diabetic wounds by RNA interference (RNAi) therapy (Huang et al., 2021b). Moreover, to obtain stem cell exosomes loaded with HOX transcript antisense lncRNA (HOTAIR), MSCs were transfected to overexpress these lncRNAs to promote wound healing (Born et al., 2022). This indicated that the parent cells were modified and produced exosomes loaded with mRNA and proteins, which promoted wound healing. However, this method has various limitations, such as variable transfection efficiency and gene expression. Moreover, hydrophilic or hydrophobic molecules can be loaded on exosomes by co-incubating them with parent cells to improve the therapeutic effect of exosomes. One study showed that exosomes have PD-L1 on their surface after cell stimulation with IFN-γ, which affects the immunosuppressive function of recipient cells (Su et al., 2019). Exosomes carrying nucleic acids can also be derived by processing physical materials. These processes include treating BMSCs with magnetic nanoparticles (NPs) and static magnetic fields to produce exosomes containing overexpressed miRNA 1260a (miR-1260a) (Wu et al., 2021). Exosomes directly produced by parent cells have advantages in targeting and therapeutic effects. However, their disadvantages include low yield and the presence of impurities, and they need to be characterized before use.

### Exosome loading

Compared with parent cell modification, exosome modification has more beneficial effects. Exosome-loading methods include co-incubation, electroporation, high- and low-temperature cycling, and ultrasound (Nasiri Kenari et al., 2020). Cargo co-incubation is a common method. By incubating with curcumin, exosomes loaded with small anti-inflammatory molecules improve their therapeutic effect on inflammation (Sun et al., 2010). This method increases drug solubility and utilization but is limited by its low drug loading rate and unsuitability for hydrophobic molecules. Electroporation is another commonly used method for loading exosomes that has a high cargo loading rate. Moreover, electroporation increases the amount of RNA and small hydrophilic molecules loaded in exosomes, which decreases RNA degradation in the wound microenvironment (Fuhrmann et al., 2015). The therapeutic effect of functional RNA is improved by loading miRNA 21-5p (miR-21-5p) into adipose stem cell exosomes *via* electroporation, resulting in engineered exosomes with good promotive effects on diabetic wound healing (Lv Q. et al., 2020). Electroporation can produce exosomes with gene delivery and load large molecular compounds. However, it is limited to hydrophilic compounds and length-dependent gene delivery (Lamichhane et al., 2015). Considering that electroporation can lead to the aggregation of exosomes, exogenous cargos can be loaded into exosomes *via* ultrasound (Johnsen et al., 2016). Exosomes derived from HUC-MSCs were treated with ultrasound to carry silver NPs (AgNPs), which improved their antibacterial activity (Qian et al., 2020). Treatment with ultrasound maintains exosome stability and promotes cargo loading better than co-incubation (Haney et al., 2015). It is a beneficial method because of its improved gene and drug delivery. However, its disadvantages include low rates of cargo loading.

### Exosome surface modification

Exosome surface modification can improve their targeting abilities, which can be mainly achieved by genetic engineering and chemical modification (Salunkhe et al., 2020). Genetic engineering is effective for displaying genetically engineered proteins on the exosome surface, which requires exosome identification (Wan et al., 2017; Mishra et al., 2021). A previous study showed that targeted peptides can be loaded onto the exosome surface by genetic engineering, which allows tissue specificity and improves efficacy (Curley et al., 2020). Coupling cardiac stem cell exosomes with a targeted heart-homing peptide improves the targetability and uptake of exosomes in myocardial infarcted hearts (Vandergriff et al., 2018). Chemical modifications can load various molecules onto the exosome surfaces *via* non-covalent or covalent interactions. This has the advantages of fast reaction, high specificity, and water buffer compatibility (Smyth et al., 2014; Armstrong et al., 2017). Exosome surface modification can improve exosome targeting. However, it is limited by the strict separation methods that are required to obtain engineered exosomes with high purity.

### Artificial synthesis of exosomes

Artificial synthesis includes both top-down and bottom-up methods. In top-down methods, parent cells are treated to obtain vesicle-forming membrane segments, which are reassembled into exosomes. One study showed that passing ESCs through tiny pores created the derived exosomes promoting fibroblast proliferation, which contributed to tissue recovery and wound healing (Jeong et al., 2014). However, exosomes derived from cell-disrupted phospholipid bilayer membranes have donor cell signaling compounds and lipid-induced toxicity (Lv et al., 2006). The yield of artificial synthesis exosomes is higher than naturally occurring exosomes. However, their immunogenicity is lower, and they lack cargo loading specificity. Bottom-up methods can be used to prepare bilayer structures and simulate exosome components by physical or chemical methods. The lipid bilayer can then be combined with surface proteins to simulate exosome production (Vazquez-Rios et al., 2019). A previous study showed that biologically active anti-inflammatory proteins, namely APO2L and TRAIL, can be combined with liposomes to obtain artificial exosomes (Martinez-Lostao et al., 2010). Artificially synthesized stem cell exosomes have categorical characterization and composition and a controllable production process.

Compared with naturally produced exosomes, engineered exosomes have improved targeting, high drug loading rate, high purity, and large yield. Engineered exosomes with different loading cargoes can be used to promote wound healing *via* anti-inflammatory, growth-promoting, angiogenesis-promoting, and collagen synthesis-regulating activities.

## Engineered stem cell exosomes promote oral and maxillofacial wound healing

Engineered stem cell exosomes have good efficacy in promoting oral and maxillofacial wound healing by regulating inflammation, promoting fibroblast proliferation, improving angiogenesis, and decreasing scar formation.

### Engineered stem cell exosomes decrease the levels of inflammatory factor

**FIGURE 2:**
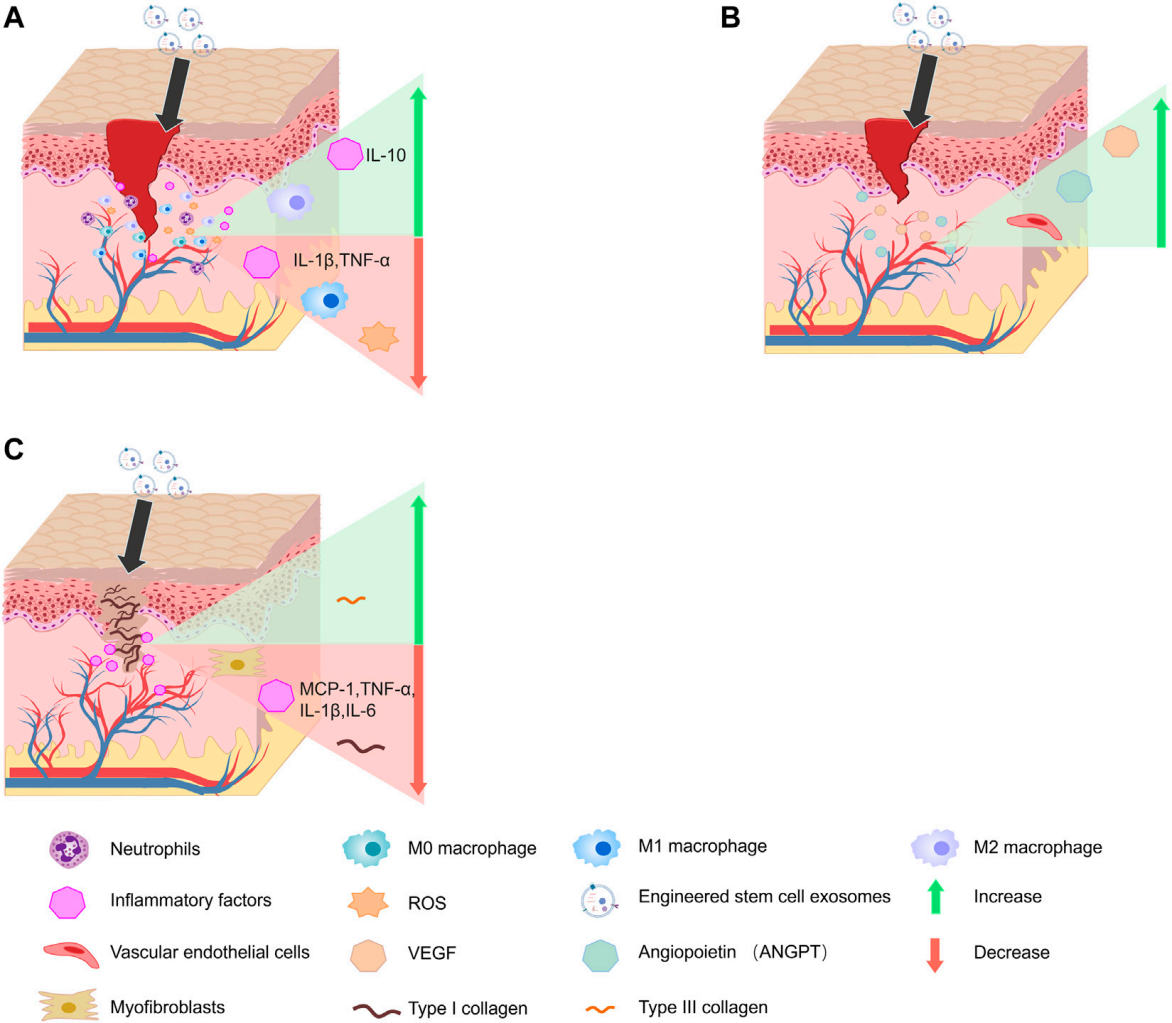
Effect of engineered stem cell exosomes on wound healing. **(A)** Engineered stem cell exosomes decrease the levels of inflammatory factor. **(B)** Engineered stem cell exosomes promote angiogenesis. **(C)** Engineered exosomes decrease scar formation.

Long-term inflammation seriously affects the wound-healing process, and sustained local inflammation can lead to abnormal wound healing and pathological scar formation (Wang et al., 2020). Engineered stem cell exosomes can decrease the inflammatory response and promote wound healing by inhibiting pro-inflammatory factor secretion (Figure 2A). One study showed that exosomes produced by treating MSCs with melatonin (MT) or deferoxamine targeted the phosphatase and tensin homolog (PTEN)/AKT signaling pathway to promote diabetic wound healing by shortening inflammatory period (Ding et al., 2019; Liu et al., 2020). Another study has shown that exosomes from MSCs stimulated with inflammatory factors such as tumor necrosis factor (TNF-α) and interferon (IFN)-γ can decrease the release of pro-inflammatory cytokines and have improved anti-inflammatory abilities (Harting et al., 2018). A previous study showed that exosomes from MSCs that are pretreated with lipopolysaccharide can regulate the TLR4/NF-κB/STAT3/AKT signaling pathway *via* miRNA let-7b to promote diabetic skin wound healing by regulating chronic inflammation regression (Ti et al., 2015). The overexpressed RNA and transcription factors of engineered stem-cell-derived exosomes play a role in promoting wound healing (Hu et al., 2019). Of note, when the transcription factor nuclear factor E2-related factor 2 (Nrf2) was overexpressed in adipose stem-cell-derived exosomes, it inhibited the production of reactive oxygen species and inflammatory cytokines to promote diabetic wound healing (Li et al., 2018). Exosomes loaded with overexpressed miRNA 181c (miR-181c) derived from HUC-MSCs decreased TNF-α and interleukin (IL)-1β and increased IL-10 levels *via* the TLR4 signaling pathway to promote burn wound healing (Li et al., 2016). Overall, the cargo of engineered stem cell exosomes can decrease the release of inflammatory factors and the duration of the inflammatory response to facilitate wound healing.

### Engineered stem cell exosomes promote fibroblast proliferation

Fibroblasts are important effector cells in skin wounds, whose function can be increased to promote wound healing by using engineered stem cell exosomes (Bhattacharyya et al., 2013; Lian and Li, 2016). A previous study showed that hypoxic adipose stem cell exosomes promoted fibroblast proliferation and migration and accelerated high-quality diabetic wound healing by activating the PI3K/AKT pathway to regulate the expression of various growth factors (Wang et al., 2021b). Moreover, engineered stem cell exosomes carrying H19 affected the H19/miR-152-3p/PTEN axis, regulating the PI3K/AKT signaling pathway to increase fibroblast proliferation and apoptosis inhibit, accelerating the diabetic wound healing process (Li B. et al., 2020). In addition, Wnt4 delivered by MSC-derived exosomes promoted β-catenin nuclear translocation and activity to increase skin cell proliferation. This played a vital role in wound re-epithelialization (Zhang et al., 2015a). Interestingly, synthetically engineered exosomes, which are effectively taken up by recipient cells, can be used for promoting wound healing. One study showed that nanovesicles obtained from extruded embryonic stem cells (ESCs) activated the mitogen-activated protein kinase (MAPK) signaling pathway to promote fibroblast proliferation and wound healing (Jeong et al., 2014). Thus, engineered stem cell exosomes can help in promoting fibroblast proliferation to accelerate wound healing through their cargos.

### Engineered stem cell exosomes promote angiogenesis

Angiogenesis is an intrinsic repair pathway for wound healing and tissue regeneration. Insufficient angiogenesis, which involves genes and proteins, such as vascular endothelial growth factor (VEGF), delays the wound-healing process (Zhao et al., 2017). Therefore, the VEGF receptor (VEGFR) can initiate angiogenesis and promote wound healing (Johnson and Wilgus, 2014). Gene-modified parental cells have been widely used to obtain exosomes that can promote angiogenesis. The proangiogenic ability of vascular endothelial cells can be increased by miRNAs and cytokines uptake by engineered stem cell exosomes (Figure 2B). On study showed that MSC exosomes with high levels of miRNA 126-3p (miR-126-3p) can promote human dermal microvascular endothelial cell proliferation, migration, and angiogenesis (Tao et al., 2017). MSC exosomes carrying miR-21-5p promote angiogenesis by upregulating the VEGF, AKT, and MAPK pathways, which positively affects diabetic wound healing (Huang C. et al., 2021). Moreover, the vascularization ability of exosomes can also be increased by physical processing. One study showed that blue light treatment upregulated miR-135b-5p and miR-499a-3p levels in HUC-MSC-derived exosomes, which promoted human umbilical vein endothelial cell (HUVECs) proliferation, migration, and angiogenesis (Yang et al., 2019). Moreover, exosomes secreted by stem cells that are treated with various biological compounds also have significant angiogenesis-promoting abilities (Liu et al., 2019). A previous study showed that exosomes from BMSCs stimulated with iron oxide (Fe3O4) NPs and static magnetic field promoted angiogenesis by upregulating miR-21-5p targeting SPRY2 and activating PI3K/AKT and ERK1/2 signaling pathways in wound healing (Wu et al., 2020). Exosomes of BMSCs pretreated with deferoxamine activated the PI3K/AKT signaling pathway *via* miR-126-mediated PTEN downregulation to stimulate angiogenesis *in vitro* (Ding et al., 2019). Moreover, exosomes treated with superparamagnetic ferric oxide NPs have precise targeting, which accumulates in the damaged area and significantly increases angiogenesis (Li X. et al., 2020). Moreover, chemically-treated stem cell exosomes also promote angiogenesis. Stem cell exosomes pretreated with atorvastatin (ATV) or pioglitazone significantly promoted the angiogenesis of endothelial cells by mediating the PTEN/AKT/eNOS pathway in diabetic wound healing (Yu et al., 2020; Hu et al., 2021). Artificially synthesized exosomes with specific protein composition and RNA load significantly promoted angiogenesis (Tao et al., 2018; Kim et al., 2021). These findings suggested that engineered stem cell exosomes have potential therapeutic effects in promoting wound healing by modulating the proangiogenic ability of endothelial cells.

### Engineered exosomes decrease scar formation

Chronic inflammation and myofibroblast aggregation can cause the thickening of pathological scarring in the wound area (Ogawa, 2017; Rippa et al., 2019). The application of engineered stem cell exosomes can decrease scar formation (Figure 2C). A studies has shown that miRNAs 21 (miR-21), 23A (miR-23A), 125b (miR-125b), and 145 (miR-145) in MSC exosomes can inhibit fibroblast and myofibroblast differentiation by targeting TGF-β/Smad2 signaling pathway to decrease scarring (Fang et al., 2016). Moreover, human adipose-derived MSC exosomes overexpressing miRNA 29a (miR-29a) inhibit scar hyperplasia after burn injury by targeting TGF-β2/Smad3 signaling pathway (Yuan et al., 2021). Notably, engineered stem cell exosomes can inhibit scar formation by decreasing inflammatory factor expression. Previous studies have shown that human amniotic fluid stem cell exosomes can decrease scar formation by decreasing the secretion of inflammation-related cytokines *via* miRNA 146a-5p (miR-146a-5p) (Wgealla et al., 2022). Exosomes derived from MSCs overexpressing tumor necrosis factor (TNF)-stimulated gene-6 (TSG-6) can decrease MCP-1, TNF-α, IL-1β, and IL-6 levels in scar tissue and inhibit the inflammatory response in pathological scars, significantly reducing scar formation (Jiang et al., 2020). Therefore, engineered stem cell exosomes are an effective approach for enhancing their biological activity and improving repair efficacy in reducing scar formation.

### The application of engineered exosomes in oral and maxillofacial wound

Oral and maxillofacial injury can easily damage the hard and soft tissues (Lv L. et al., 2020). Because of the special location of oral and maxillofacial, the healing process has an important effect on the physiological function and mental health of patients. The application of engineered exosomes can positively promote the accurate and efficient healing of oral and maxillofacial wounds. Engineered exosomes can increase accumulation at the wound site by precise delivery, thereby effectively promoting wound healing (Li X. et al., 2020). Furthermore, the engineered exosomes can achieve a therapeutic effect on oral and maxillofacial wound healing through their cargos, including diabetes and burn wounds, which are difficult to heal (Aryan et al., 2018; Peng Q. et al., 2020; Liu et al., 2021; Hade et al., 2022; Hsu et al., 2022). Moreover, because oral and maxillofacial nerve repair is directly associated with expression, the application of engineered exosomes can contribute to nerve repair and regeneration and promote the early recovery of expression functions (Yang et al., 2021; Wang Y. et al., 2022). Compared with traditional treatment and cell therapy, engineered stem cell exosomes have better effects on oral and maxillofacial wound healing and have good application prospects (Maqsood et al., 2020; Md Fadilah et al., 2022). Presently, many more efficient and safer cell-free scaffold dressings carrying exosomes have been developed for wound healing (Las Heras et al., 2020). The combined application of exosomes and biomaterials has a positive effect on promoting wound healing, which can maintain the stability of exosomes *in vivo* with good biocompatibility (Golchin et al., 2022).

## The application of exosomes in clinical trials

Stem cell-derived exosomes avoid the risk of cell therapy and have a good application prospect in regenerative medicine (Phinney and Pittenger, 2017). The clinical application of exosomes in wound healing is gradually increasing (Supplementary Table S1) (Li et al., 2022). One study evaluated the effectiveness of exosomes extracted from adipose tissue of patients for wound healing (NCT05475418). In an early Phase 1 clinical trial, exosomes extracted from the plasma of patients were applied to the ulcer site to clear the effectiveness of exosomes in promoting skin wound healing (NCT02565264). In addition, a clinical trial started in March 2022 has completed the safety and tolerability study of the topical application of MSC exosome ointment in Psoriasis (NCT05523011). Moreover, a clinical study on atrophic acne scar treatments using adipose tissue-derived stromal cells (ADSCs) exosomes showed promising therapeutic effects (Kwon et al., 2020).

With the advantages of good biocompatibility and low immunogenicity, exosomes provide new tools for the development of therapeutic drugs for human wound healing (Hade et al., 2021). Moreover, exosomes can protect the cargos they carry by a biomembrane from enzymes and other substances that can damage proteins, and exosome-packaged proteins are more stable and efficient (Lu et al., 2019). Therefore, oral and maxillofacial trauma can be treated by synthesizing engineered exosomes with specific functions. At present, with the in-depth study of engineered exosome preparation technology and treatment mechanism, some exosome-based drugs have been developed, and the clinical application of exosomes still has a broad space to explore.

## Discussion

Presently, the application of hydrogel-loaded and ECM-loaded stem cells has positively helped in the process of wound healing (Lee et al., 2007; Rustad et al., 2012; Ariyanti et al., 2019). Stem cells play a crucial role in regenerating damaged organs *via* their paracrine effects (Ratajczak et al., 2012). Because the knowledge of intercellular functions has increased, exosomes can be developed as a precise and targeted therapeutic strategy (van Niel et al., 2018). Compared with normal cells, stem-cell-derived exosomes can escape phagocytosis and have the advantages of greater biocompatibility, increased retention, and low immunogenicity (Saunderson et al., 2014; Kamerkar et al., 2017; Wortzel et al., 2019; Hassanzadeh et al., 2021). Importantly, stem-cell-derived exosomes have the advantages of easier storage and application, penetration of the blood–brain barrier, and a long circulation half-life (Turturici et al., 2014; Chen et al., 2016; Gowen et al., 2020; Huang et al., 2021c). The function of exosomes is related to the donor cells and environmental stimuli (Stavrou and Ortiz, 2022). Stem cells and exosomes in adipose tissue have a positive impact on promoting angiogenesis, which has great application potential in regenerative medicine (Kamat et al., 2020). Compared with stem cells derived from other tissues, mesenchymal stem cells in adipose tissue are easily accessed and have a high density (Buschmann et al., 2013). Moreover, a study shows that microfragmented adipose tissue (MFAT) has long-lasting anti-inflammatory activity (Nava et al., 2019). Clinical trials proved that MFAT used to treat ulcer caused by prosthesis effectively promote rapid healing of skin ulcers (Copeland and Martin, 2021). Therefore, stem-cell-derived exosomes have beneficial advantages in wound healing and can be potentially used in wound healing therapies.

Exosomes can be used as a delivery system for drugs, proteins, mRNA, miRNA, lncRNA, and small molecules to recipient cells, which can regulate macrophages, fibroblasts, vascular endothelial cells, and myofibroblast functions in wounds to promote healing (Bunggulawa et al., 2018). They promote the uptake by recipient cells and protect RNA from extracellular degradation, thereby enabling them to be an ideal treatment tool for oral and maxillofacial wound healing (Kooijmans et al., 2012; Zhang et al., 2013; Zhang J. et al., 2015).

**FIGURE 3:**
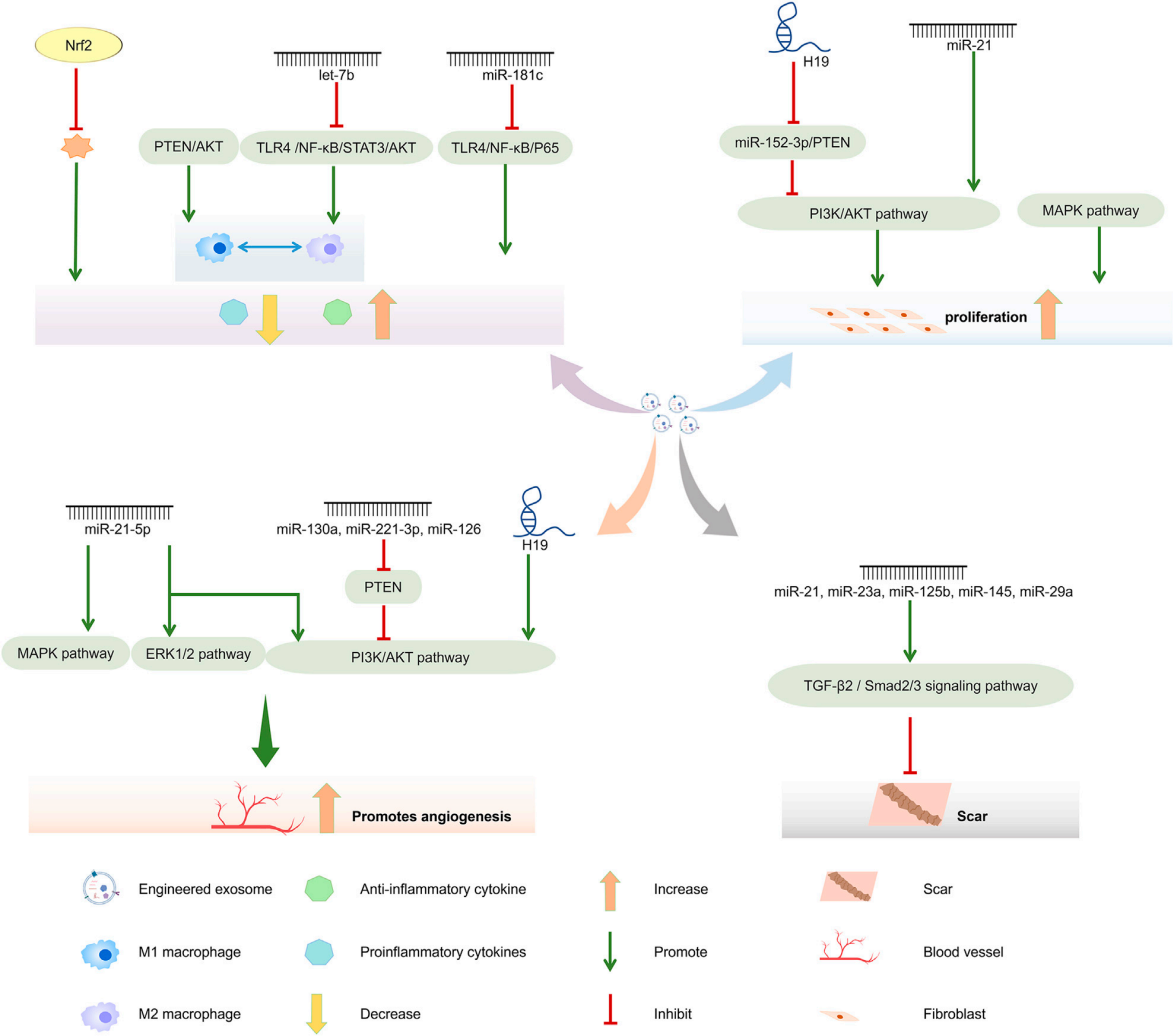
Wound healing promoting mechanism of engineered stem cell exosomes.

However, the application of naturally produced exosomes is limited (Villarroya-Beltri et al., 2014; Taylor and Shah, 2015; Bjorge et al., 2017; Bian et al., 2022). Nevertheless, engineered stem cell exosomes loaded with functional cargos can have remarkable therapeutic effects (Marcus and Leonard, 2013). The exosomes are loaded with overexpressed nucleic acids by various methods, such as transfection, co-incubation, ultrasound, and electroporation, which provide them with targeting, anti-inflammatory, cell proliferation, and apoptotic functions (Weaver, 1993; Jo et al., 2014; Smyth et al., 2014; Fang et al., 2016; Garcia-Manrique et al., 2018; Ding et al., 2019; Li B. et al., 2020; Li X. et al., 2020). Importantly, engineered exosomes can be designed for different wound types, such as diabetes and burns, with functions in attenuating anti-inflammatory processes, promoting angiogenesis, and decreasing scar formation (Lv L. et al., 2020) (Figure 3). However, it is necessary to solve the needs of large-scale production and high purity for clinical application. Hollow fiber bioreactors are ideal for large-scale production (Kimiz-Gebologlu and Oncel, 2022). In recent years, with the advancement of technology, microfluidics, electrical, centrifugal and acoustical forces have been introduced into the separation of exosomes, which has led to the rapid development of some new exosome separation technologies. They include polymer-based precipitation, ultrafiltration, bioreactor systems, production of biomimetic vesicles, membrane-based separation methods, and microfluidic methods (Supplementary Table S2) (Wang et al., 2021a; Zhang et al., 2021). Among them, microfluidic devices that can be used for exosome isolation and purification become promising devices for exosome therapy (Chen et al., 2021). The bottom-up production of synthetic exosomes in engineered exosomes can be synthesized by a method similar to liposome microfluidic to realize large-scale production of exosomes (Wang X. et al., 2022), which will promote the application of exosomes in oral and maxillofacial wound healing.

The oral and maxillofacial region contains various important blood vessels and nerves that are vulnerable to trauma and prone to bleeding and infection after any injury (Kageyama et al., 2021). The engineered stem cell exosomes can act as biological carriers delivering various bioactive substances to act on target cells. They can participate in the regulation of wound repair *via* signal transduction, which can be effective in oral and maxillofacial wound healing (Maqsood et al., 2020).

## Conclusion

Engineered stem cell exosomes have high yields, low immunogenicity, and specific functional cargos. Moreover, engineered stem cell exosomes can decrease inflammation and scar formation and promote angiogenesis and fibroblast proliferation. These properties of engineered stem cell exosomes can be leveraged in the future for oral and maxillofacial wound healing.
