# Supplementary material for: Engineered stem cell exosomes for oral and maxillofacial wound healing
Source: Front Bioeng Biotechnol. 2022 Oct 24;10:1038261. doi: 10.3389/fbioe.2022.1038261 (PMC9637828; doi:10.3389/fbioe.2022.1038261)
Supplement: Supplementary file 1 [file DataSheet1.docx]

**Table S1: Clinical application of exosomes to wound healing.**

| Condition or Disease | Phase | Origin of Exosomes | Research Purpose | First Posted Time | Results | References /ClinicalTrials.gov Identifier |
| --- | --- | --- | --- | --- | --- | --- |
| Wounds and Injuries | Not Applicable | subject adipose tissue | Treatment | July 26, 2022 | Not yet recruiting | NCT05475418 |
| Ulcer | Early Phase 1 | autologous plasma | Treatment | October 1, 2015 | No Results Posted | NCT02565264 |
| Dystrophic Epidermolysis Bullosa | phase 1/2A | AGLE-102 (allogeneic derived extracellular vesicle) | Treatment | November 22, 2019 | Not yet recruiting | NCT04173650 |
| Psoriasis | Phase 1 | MSC exosome ointment | Treatment | August 31, 2022 | Completed | NCT05523011 |
| Atrophic acne scar | Clinical trial | adipose tissue stem cell | Treatment |  | Application of adipose tissue stem cell-derived exosomes treatment yielded more favourable responses, a shorter recovery time, and fewer side- effects. | (1) |

| Large-Scale Production Technique | Principle | Potential advantages | Potential disadvantages | References |
| --- | --- | --- | --- | --- |
| Microfluidics-  based techniques | Surface antigens; Density; Size; Acoustic field; Magnetic field; Electric field. | Continuous-flow; Biocompatibility; Label-free; Portability; Reproducibility; High specificity, throughput, and efficiency; Consume low volumes of sample and reagent; High purity. | Moderate to low sample capacity; Need method validation and standardization. | (2-5) |
| Membrane-based Isolation | Phospholipid bilayer membrane properties. | High efficiency; High affinity; Rapid. | Low purity; Contain other impurities with membrane. | (4, 6, 7) |
| Polymer-based Precipitation | Solubility; Surface charge. | Easily scale up; High yield; Fast and easy application; Low labour required; Commercial kits available. | Low purity; Need for extra purification steps; Containing free highly heterogeneous and protein-contaminated exosomes mixture. | (8, 9) |
| Ultrafiltration | Size; Molecular weight. | Rapid; Not require special equipment. | Pore clogging; Vesicle trapping; Low isolation efficiency; Low purity; Exosome loss due to attaching to the membranes; Can result in deformation or damage of exosomes. | (3, 10) |
| Bioreactor Systems | Cell culture flasks or bioreactors. | High yield; Homogeneity; Controllability. | Cell aging; Low purity; The production system and mode used need to be optimized for each cell line. | (11-13) |
| Production of Biomimetic Vesicles | Self-assembly of membrane vesicles | Lower labor and time costs; Homogeneous size; High yield; Homogeneous composition. | Inability to recycle cells; The loss of cytoplasmic content; The contamination of vesicle preparation with nuclear components; Must undergo a biological activity evaluation. | (14) |

**Table S2: Methods for large-scale production of exosomes.**

**References**

1. Kwon HH, Yang SH, Lee J, Park BC, Park KY, Jung JY, et al. Combination Treatment with Human Adipose Tissue Stem Cell-Derived Exosomes and Fractional Co2 Laser for Acne Scars: A 12-Week Prospective, Double-Blind, Randomized, Split-Face Study. *Acta Derm Venereol* (2020) 100(18):adv00310. doi: 10.2340/00015555-3666.

2. Shirejini SZ, Inci F. The Yin and Yang of Exosome Isolation Methods: Conventional Practice, Microfluidics, and Commercial Kits. *Biotechnol Adv* (2022) 54:107814. Epub 20210811. doi: 10.1016/j.biotechadv.2021.107814.

3. Li P, Kaslan M, Lee SH, Yao J, Gao Z. Progress in Exosome Isolation Techniques. *Theranostics* (2017) 7(3):789-804. Epub 20170126. doi: 10.7150/thno.18133.

4. Chen J, Li P, Zhang T, Xu Z, Huang X, Wang R, et al. Review on Strategies and Technologies for Exosome Isolation and Purification. *Front Bioeng Biotechnol* (2021) 9:811971. Epub 20220105. doi: 10.3389/fbioe.2021.811971.

5. Wang J, Ma P, Kim DH, Liu BF, Demirci U. Towards Microfluidic-Based Exosome Isolation and Detection for Tumor Therapy. *Nano Today* (2021) 37. Epub 20210113. doi: 10.1016/j.nantod.2020.101066.

6. Zhang N, Sun N, Deng C. Rapid Isolation and Proteome Analysis of Urinary Exosome Based on Double Interactions of Fe3o4@Tio2-DNA Aptamer. *Talanta* (2021) 221:121571. Epub 20200901. doi: 10.1016/j.talanta.2020.121571.

7. Kim H, Shin S. Exocas-2: Rapid and Pure Isolation of Exosomes by Anionic Exchange Using Magnetic Beads. *Biomedicines* (2021) 9(1). Epub 20210102. doi: 10.3390/biomedicines9010028.

8. Zhu L, Sun HT, Wang S, Huang SL, Zheng Y, Wang CQ, et al. Isolation and Characterization of Exosomes for Cancer Research. *J Hematol Oncol* (2020) 13(1):152. Epub 20201110. doi: 10.1186/s13045-020-00987-y.

9. Kotmakçı M. *Exosome Isolation: Is There an Optimal Method with Regard to Diagnosis or Treatment?* sine loco: IntechOpen (2017).

10. Inamdar S, Nitiyanandan R, Rege K. Emerging Applications of Exosomes in Cancer Therapeutics and Diagnostics. *Bioeng Transl Med* (2017) 2(1):70-80. Epub 20170403. doi: 10.1002/btm2.10059.

11. Colao IL, Corteling R, Bracewell D, Wall I. Manufacturing Exosomes: A Promising Therapeutic Platform. *Trends Mol Med* (2018) 24(3):242-56. Epub 20180212. doi: 10.1016/j.molmed.2018.01.006.

12. Patel DB, Santoro M, Born LJ, Fisher JP, Jay SM. Towards Rationally Designed Biomanufacturing of Therapeutic Extracellular Vesicles: Impact of the Bioproduction Microenvironment. *Biotechnol Adv* (2018) 36(8):2051-9. Epub 20180912. doi: 10.1016/j.biotechadv.2018.09.001.

13. Paganini C, Capasso Palmiero U, Pocsfalvi G, Touzet N, Bongiovanni A, Arosio P. Scalable Production and Isolation of Extracellular Vesicles: Available Sources and Lessons from Current Industrial Bioprocesses. *Biotechnol J* (2019) 14(10):e1800528. Epub 20190708. doi: 10.1002/biot.201800528.

14. Syromiatnikova V, Prokopeva A, Gomzikova M. Methods of the Large-Scale Production of Extracellular Vesicles. *Int J Mol Sci* (2022) 23(18). Epub 20220910. doi: 10.3390/ijms231810522.
